# Prognostic value of cell-free plasma DNA in patients with cardiac arrest outside the hospital: an observational cohort study

**DOI:** 10.1186/cc8934

**Published:** 2010-03-29

**Authors:** Francisco Arnalich, Marta Menéndez, Verónica Lagos, Enrique Ciria, Angustias Quesada, Rosa Codoceo, Juan José Vazquez, Eduardo López-Collazo, Carmen Montiel

**Affiliations:** 1 Emergency Medicine Department, Internal Medicine Service, Hospital Universitario La Paz, IDIPaz. Paseo de la Castellana 261. 28046 Madrid, Spain; 2 Department of Pharmacology and Therapeutics, IDIPaz, Facultad de Medicina, Universidad Autónoma de Madrid. Arzobispo Morcillo, 4. 28029 Madrid. Spain; 3 Clinical Biochemistry Service, Hospital Universitario La Paz, IDIPaz. Paseo de la Castellana 261. 28046 Madrid, Spain; 4 Medical Research Unit. Hospital Universitario La Paz, IDIPaz Paseo de la Castellana 261. 28046 Madrid, Spain

## Abstract

**Introduction:**

Many approaches have been examined to try to predict patient outcome after cardiopulmonary resuscitation. It has been shown that plasma DNA could predict mortality in critically ill patients but no data are available regarding its clinical value in patients after out-of-hospital cardiac arrest. In this study we investigated whether plasma DNA on arrival at the emergency room may be useful in predicting the outcome of these patients.

**Methods:**

We performed a prospective study of out-of-hospital patients with cardiac arrest who achieved return of spontaneous circulation after successful resuscitation. Cardiovascular co-morbidities and resuscitation history were recorded according to the Utstein Style. The outcome measures were 24 h and overall in-hospital mortality. Cell-free plasma DNA was measured by real-time quantitative PCR assay for the β-globin gene in blood samples drawn within two hours after the arrest. Descriptive statistics, multiple logistic regression analysis, and receiver operator characteristic (ROC) curves were calculated.

**Results:**

Eighty-five consecutive patients were analyzed with a median time to return of spontaneous circulation of 27 minutes (interquartile range (IQR) 18 to 35). Thirty patients died within 24 h and 58 died during the hospital course. Plasma DNA concentrations at admission were higher in non-survivors at 24 h than in survivors (median 5,520 genome equivalents (GE)/ml, vs 2810 GE/ml, *P *< 0.01), and were also higher in patients who died in the hospital than in survivors to discharge (median 4,150 GE/ml vs 2,460 GE/ml, *P *< 0.01). Lactate clearance at six hours was significantly higher in 24 h survivors (*P *< 0.05). The area under the ROC curves for plasma DNA to predict 24-hour mortality and in-hospital mortality were 0.796 (95% confidence interval (CI) 0.701 to 0.890) and 0.652 (95% CI 0.533 to 0.770). The best cut-off value of plasma DNA for 24-h mortality was 4,340 GE/ml (sensitivity 76%, specificity 83%), and for in-hospital mortality was 3,485 GE/ml (sensitivity 63%, specificity 69%). Multiple logistic regression analysis showed that the risk of 24-h and of in-hospital mortality increased 1.75-fold and 1.36-fold respectively, for every 500 GE/ml increase in plasma DNA.

**Conclusions:**

Plasma DNA levels may be a useful biomarker in predicting outcome after out-of hospital cardiac arrest.

## Introduction

Overall survival rate from out-of-hospital cardiac arrest has not increased in parallel with the improvements in cardiopulmonary resuscitation (CPR) [1,2]. The hospital discharge rate is 15% in a meta-analysis that included a total population of over 26,000 patients [3]. Pre-morbid factors, peri-arrest and post-arrest variables [4,5], and several serum markers, for example, two neuroproteins, neuro-specific enolase and S-100 [6,7], serum lactate [8,9], and B-type natriuretic peptide [10,11] have been examined to predict outcome after CPR, although none have proved entirely useful.

The majority of patients who achieve return of spontaneous circulation after successful CPR have a high risk to death in the post-arrest period. A few clinical studies have shown elevated plasma concentrations of soluble adhesion molecules (selectins) [12] and cytokines [13,14] in patients resuscitated from cardiac arrest. This immediate post-resuscitation period has some similarities to the sepsis syndrome and septic shock in terms of the inflammatory cascade activation and microcirculatory hypoperfusion [15]. As increased concentrations of cell-free DNA have been found in patients with sepsis and septic shock [16-18], and the plasma DNA concentration is an independent predictor for ICU mortality in these patients [19], we hypothesized that admission DNA concentrations may also predict mortality in patients in the post-cardiac arrest resuscitation period. Therefore, the aim of this study was to evaluate whether cell-free plasma DNA on admission is associated with short-term mortality in patients after out-of-hospital cardiac arrest.

## Materials and methods

### Patients and setting

Between January 2005 and June 2007, 113 consecutive adult patients who presented to the emergency room after non-traumatic, normothermic, out-of-hospital cardiac arrest were recruited into the study. The inclusion criteria were: 1) age more than 17 years, 2) cardiac arrest prior to the arrival of emergency personnel, 3) pre-arrest GCS = 15 or independent ADLs, 4) no written *do not attempt resuscitation *(DNAR) order. Exclusion criteria were: 1) successful resuscitation by bystanders prior to arrival of pre-hospital providers, 2) interval between collapse and the start of CPR longer than 15 minutes, 3) no return of spontaneous circulation could be achieved within 60 minutes, 4) survival for less than 12 hours after the event, 5) chronic renal failure treated by hemodialysis, neoplastic diseases, stroke or acute coronary syndrome in the previous 30 days, 6) the emergency physician was unable to diagnose their disease, and 7) their families refused to provide informed consent to participate. The study was approved by the local ethics committee. Patient data were collected according to the Utstein Style [20,21] in which cardiac arrest is defined as the absence of palpable pulse and effective spontaneous respiration with initial rhythm ventricular fibrillation (VF), pulseless ventricular tachycardia (PVT), pulseless electrical activity (PEA) and asystole. Resuscitation protocols followed the European Resuscitation Council guidelines [22] and the American Heart Association guidelines [23,24]. Therapeutic hypothermia (33°C as the target temperature for 24 h) was subsequently performed in comatose survivors whose systolic blood pressure had increased to above 90 mm Hg [25,26]. The primary endpoint in the study was 24-h mortality. Secondary endpoint was in-hospital mortality.

### Blood sampling, processing of plasma and DNA extraction

After return of spontaneous circulation with standard advanced cardiovascular life support according to the European Resuscitation Council guidelines [22] and the American Heart Association guidelines [23,24], a 10 ml blood sample to measure cell-free plasma DNA was taken from the antecubital vein of each patient immediately after return of spontaneous circulation in the emergency room. Plasma and cells were separated by centrifugation at 1,600 g (+4°C) for 10 minutes and plasma samples were stored at -80°C. Plasma samples were centrifuged at 16,000 g for 10 minutes before DNA extraction to remove any residual cells. DNA was extracted from 200-μl plasma samples using the QIAamp DNA Blood Mini Kit (QIAGEN, Hilden, Germany) according to the *blood and body fluid *protocol recommended by the manufacturer.

### Real-time quantitative PCR

Plasma DNA was measured in duplicate samples by real-time quantitative PCR assay for the β-globin gene [27] using the ABI PRISM 7000 sequence detection system (Applied Biosystems Inc, Foster City,, CA 94404, USA). PCR primers and the fluorescent probe were designed by Primer Express software (Applied Biosystems). The primer and probe sequences were as follows: forward primer 5'-GCA CCT GAC TCC TGA GGA GAA-3', reverse primer 5'-CAC CAA CTT CAT CCA CGT TCA-3', and a single-labeled fluorescent MGB-probe 5'-FAM-TCT GCC GTT ACT GCC CT-MGB-NFQ, where MGB is a minor groove binding molecule and NFQ a non-fluorescent quencher molecule. Samples were analyzed in duplicate in a reaction volume of 25 μl containing 5 μl of sample, 300 nM of each primer, 200 nM of probe and 1×Taqman master mix (Applied Biosystems). PCR cycling conditions were two minutes at +50°C, 10 minutes at +95°C, and 46 cycles of 20 seconds at +95°C and one minute at +60°C. We used a 10-fold serial dilution of human genomic DNA (QIAGEN, Hilden, Germany) as a standard curve. The imprecision of this system has been reported previously (20), with a CV for the threshold cycle of 1.1%. Raw data are converted into units of copies of genomes, and expressed as genome equivalents (GE), per ml plasma (1 GE = 6.6 pg DNA). DNA levels are given to the nearest 25 genome-equivalents (GE)/ml and the detection limit is 12.5 GE/ml.

### Statistics

Continuous data are presented as the median and interquartile range. Discrete variables are given as counts and percentages. Lactate clearance at six hours was defined as the difference in initial lactate concentration on arrival at the ED to six hours afterwards divided by initial lactate concentration value and multiplied by 100. Univariate comparisons of continuous data were performed by Mann-Whitney *U*-test, and by Chi-square for categorical variables. Non-normal distributions were transformed into normal distributions using a logarithmic transformation. The confidence interval (95% CI) was determined as an indication of the precision of an estimate of a population value. The odds ratio (OR) was calculated as an estimate of relative risk between two groups on the basis of the mortality as outcome. Multiple logistic regression analysis was used to determine the independent contribution of multiple variables to the outcome of 24-h and in-hospital mortality, and for calculation of adjusted odds ratio. We selected candidate variables for the regression model that were shown to impact mortality in prior studies [1,2,26]. A *P *< 0.01 level was used for the inclusion of the variables in the model. The discriminative power of DNA and lactate clearance to predict mortality was determined with the use of receiver operator characteristic (ROC) curves. We calculated areas under the curve (AUCs) with 95% CIs, the best predictive cut-off values and positive likelihood ratios with 95% CIs according to standard procedures. Statistical significance was set at *P *< 0.05 in all tests. The statistical analyses were computed with SPSS 12.0 statistical software (SPSS, Chicago, Ill., USA).

## Results

Overall, 85 patients matched the inclusion criteria for this study. The cause of cardiac arrest was: underlying cardiac disorder (n = 46), respiratory failure (n = 30), metabolic factors (n = 6) and hypovolemia (n = 3). Twenty-four-hour mortality and in-hospital mortality were 35.2% and 65.8%, respectively (Table 1). Patient demography and medical history prior to cardiac arrest, the initial ECG-pattern, and the clinical findings at the time of admission to the emergency room are described in Table 1. Acute myocardial infarction (AMI) was determined as the final diagnosis and cause of cardiac arrest in 48 patients (56.5%), 35 patients (72.9%) had coronary angiography and 25 (52.1%) received percutaneous coronary intervention with stent placement. The main artery occluded was the left anterior descending in 14 patients, the right coronary artery in eight and the circumflex in seven. Four patients had more than one artery involved. Eighteen patients were treated with mild therapeutic hypothermia according to the ALS Task Force of the International Liaison Committee on Resuscitation (25). Initial cold fluid infusions and ice packs combined with external cooling with cold blankets was used to achieve a core temperature of 33°C (time to achievement 5.3 h ± 2.1 h) and maintained for 24 hours.

**Table 1:** Descriptive characteristics of the study cohort

Age, years		62 (51 to 69)
**Female**		38 (44.7)

**Cause of cardiac arrest**	Underlying cardiac disorder	48 (56.5)

	Respiratory failure	28 (32.9)

	Metabolic factors	7 (8.2)

	Hypovolemia	2 (2.4)

**Comorbidity**	Previous healthy	16 (18.8)

	Obesity	52 (61.2)

	Diabetes	30 (35.3)

	Hypertension	48 (56.4)

	Coronary artery disease	29 (34.1)

	Chronic heart failure	27 (31.7)

	COPD/emphysema	24 (28.2)

**Initial cardiac rhythm**	Ventricular fibrilation	19 (22.4)

	Pulseles electrical activity	21 (24.7)

	Asystole	45 (52.9)

**Resuscitation factors**	"no flow" time	3 (2 to 6)

	"low flow" time	24 (18 to 34)

	Witnessed arrest, n (%)	47 (55.2)

	Bystander CPR, n (%)	25 (29,4)

	Ongoing CPR	21 (24.7)

	Glasgow Coma Scale	6 (4 to 8)

	Defibrillation	40 (47.1)

	Intravenous adrenaline	67 (78.8)

	Adrenaline dose (mg)	4 (2 to 5)

**In-hospital diagnosis and treatment**	Head CT	82 (96.4)

	Thorax CT	37 (43.5)

	Mild therapeutic hypothermia	18 (21.2)

	Cardiogenic shock	16 (18.8)

	Acute myocardial infarction	48 (56.5)

	Coronary angiography	35 (72.9% of the AMI)

	Percutaneous coronary intervention	25 (52.1% of the AMI)

	Intra-aortic balloon pump	6 (7.1)

**Clinical outcome**	24-h mortality	30 (35.2)

	In-hospital mortality	56 (65.8)

**Laboratory values**	pH	7.14 (7.08 to 7.20)

	Basal lactate (mmol/l)	9.6 (7.0 to 12.7)

	6-h lactate (mmol/l)	6.7 (4.8 to 8.0)

	6-h lactate clearance (%)	45 (32 to 58)

	Bicarbonate (mmol/l)	12.8 (10.3 to 17.8)

	Glucose (mg/dl)	210 (175 to 240)

	Blood urea nitrogen (mg/dl)	38 (28 to 52)

	Creatinine (mg/dl)	1.5 (1.1 to 1.8)

	Troponin I (ng/dl)	1.0 (0.5 to 1.4)

Data are median (IQR) or number (%). CT, computed tomography.

The median duration of the ICU stay was 12 days (IQR 5 to 21), and the median time until hospital discharge was 36 days (IQR 19 to 47). Clinical characteristics of 24-hour survivors and non-survivors are listed in Table 2. Except for the presence of diabetes, there was no statistical difference with respect to other cardiovascular risk factors or comorbidities. The median cell-free plasma DNA concentration at admission was higher in non-survivors at 24 hours than in survivors (5,520 GE/ml, vs 2,810 GE/ml, *P *< 0.01). The plasma DNA concentration was higher in patients with CPR duration longer than 30 minutes than in patients with shorter time of resuscitation (4,470 GE/ml, vs 3,150 GE/ml, *P *< 0.05). In addition to plasma DNA, bystander basic life support, total downtime interval (time from collapse until return of spontaneous circulation), asystole as the presenting cardiac rhythm, ongoing CPR on arrival at the emergency room, palpable pulse on arrival at the emergency room, six-hour lactate concentration, six-hour lactate clearance, serum glucose and urea concentrations, and confirmed AMI as final diagnosis were also found to be predictive of 24-hour mortality in a univariate analysis (Table 2). The plasma DNA level at admission was significantly correlated with the total downtime (r = 0.579, *P *< 0.001), maximum lactate concentration (r = 0.602, *P *< 0.001), and the first 24-hour APACHE II score (r = 0.415, *P *< 0.003). Plasma DNA concentration did not correlate with urea concentration (r = 0.26, *P *= 0.053), nor was it in correlation with age, leukocyte count, troponin, creatinine or glucose.

**Table 2:** Univariate analysis: comparisons of factors associated with 24-h mortality

	Survivors (n = 55)	Non-survivors (n = 30)	*P*
Age, years	60 (51 to 69)	62 (52 to 70)	NS
			
Female,	25 (45.4)	13 (43.3)	NS
			
Hypertension	30 (54.5)	18 (60.0)	NS
			
Diabetes	16 (29.1)	14 (46.6)	<0.05
			
Coronary artery disease	18 (32.7)	11 (36.6)	NS
			
Chronic Heart Failure	16 (29.1)	11 (36.6)	NS
			
Witnessed arrest	31 (56.4)	16 (53.3)	NS
			
Bystander CPR	20 (36.4)	5 (16.6)	<0.01
			
Total downtime	22 (15 to 29)	31 ((22 to 38)	0,002
			
CGS <6 on arrival at the ER	47 (85.5)	27 (90.0)	NS
			
Ongoing CPR on arrival at the ER	16 (29.1)	5 (16.7)	<0.05
			
Palpable pulse on arrival at the ER	45 (90.9)	19 (70.0)	<0.01
			
Supraventricular rhythm in the ER	34 (61.8)	20 (66.7)	NS
			
Asystole	23 (41.8)	22 (73.3)	0.002
			
Defibrillation in the ER	27 (49.1)	13 (43.3)	NS
			
Adrenaline in the ER	41 (74.5)	26 (86.7)	NS
			
Cardiogenic shock	9 (16.4)	7 (23.3)	NS
			
Confirmed AMI as final diagnosis	34 (61.8)	14 (46.7)	<0.05
			
APACHE II score	35 (32 to 38)	38 (33 to 41)	NS
			
Plasma DNA (GE/ml)	3,970 (2,460 to 4,980)	5,520 (3,870 to 6,400)	0.001
			
Basal lactate (mmol/l)	10.7 (9.6 to 12.2)	11.9 (10.7 to 13.4)	NS
			
6-h lactate (mmol/l)	5.7 (4.9 to 6.6)	8.4 (7.1 to 10.2)	<0.01
			
6-h lactate clearance (%)	55 (47 to 65)	38 (16 to 57)	<0.05
			
Blood sugar (mg/dl)	214 (180 to 234)	255 (210 to 275)	<0.05
			
Blood urea nitrogen (mg/dl)	35 (28 to 41)	46 (33 to 54)	<0.05

Data are median (IQR) or number (%). CPR, cardiopulmonary resuscitation; CGS, coma Glasgow scale; ER, emergency room; AMI, acute myocardial infarction.

Plasma DNA concentrations at admission also showed statistical significance regarding the secondary endpoint of in-hospital mortality (Table 3). Plasma DNA concentrations were higher in hospital non-survivors than in survivors to discharge (median 4,150 GE/ml vs 2,430 GE/ml, *P *< 0.01). Asystole as the presenting cardiac rhythm and confirmed AMI as final diagnosis were also found to be statistically significant.

**Table 3:** Univariate analysis: comparisons of factors associated with in-hospital mortality

	Survivors (n = 29)	Non-survivors (n = 56)	*P*
Age, years	60 (51 to 69)	61 (52 to 70)	NS
			
Female	13 (44.8)	25 (43.3)	NS
			
Hypertension	15(51.7)	33 (58.9)	NS
			
Diabetes	12 (41.4)	30 (53.6)	NS
			
Coronary artery disease	12 (41.4)	27 (48.2)	NS
			
Chronic heart failure	9 (31.0)	18 (32.1)	NS
			
Witnessed arrest	18 (62.1)	29 (51.8)	NS
			
Bystander CPR	10 (16.9)	15 (26.8)	NS
			
Total downtime, min	20 (15 to 29)	27 (22 to 35)	NS
			
CGS < 6 on arrival at the ER	24 (82.7)	50 (89.2)	NS
			
Ongoing CPR on arrival at the ER	7 (24.1)	14 (25.0)	NS
			
Palpable pulse on arrival in the ER	23 (79.3)	48 (85.7)	NS
			
Supraventricular rhythm in the ER	17 (58.6)	37 (66.1)	NS
			
Asystole	11 (37.9)	34 (60.7)	< 0.01
			
Defibrillation in the ER	15 (51.7)	25 (44.6)	NS
			
Adrenaline in the ER	21 (72.4)	46 (82.1)	NS
			
Cardiogenic shock	5 (17.2)	11 (19.6)	NS
			
Confirmed AMI as final diagnosis	19 (65.5)	29 (51.8)	< 0.05
			
APACHE II score	34 (32 to 38)	37 (33 to 41)	NS
			
Plasma DNA (GE/ml)	2,400 (1,370 to 3,550)	4,150 (3,460 to 5,180)	0.001
			
Basal lactate (mmol/l)	10.8 (9.1 to 12.2)	11.5 (10.3 to 13.1)	NS
			
6-h lactate (mmol/l)	5.5 (4.9 to 6.6)	6.9 (5.8 to 8.4)	NS
			
6-h lactate clearance (%)	52 (47 to 65)	43 (27 to 59)	NS
			
Blood sugar (mg/dl)	195 (165 to 220)	230 (190 to 245)	NS
			
Blood urea nitrogen (mg/dl)	34 (28 to 45)	40 (33 to 57)	NS

Data are median (IQR) or number (%).

A multivariate analysis by logistic regression to identify factors having independent predictive value for 24-hour mortality and in-hospital mortality was performed. The following variables were entered: 1) age; 2) sex; 3) diabetes mellitus; 4) hypertension; 5) coronary artery disease; 6) chronic heart failure; 7) COPD/emphysema; 8) witnessed cardiac arrest; 9) bystander initiated CPR; 10) total downtime interval; 11) asystole as the presenting cardiac rhythm; 12) unconsciousness on arrival at the ER; 13) coma Glasgow scale < 6 on arrival at the ER; 14) ongoing CPR on arrival at the ER; 15) palpable pulse on arrival at the ER; 15) supraventricular rhythm in the ER; 16) defibrillation in the ER; 17) adrenaline in the ER; 18) cardiogenic shock; 19) confirmed acute myocardial infarction as final diagnosis. Plasma DNA concentrations was the only independent predictor of 24-hour mortality and in-hospital mortality, whereas all other variables were no independently associated with the outcome (Table 4).

**Table 4:** Multiple logistic regression analyses; independent predictors of 24-h and in-hospital mortality

	24 -h mortality			In-hospital mortality		
	
	Adjusted OR	95% CI	*P *value	Adjusted OR	95% CI	*P *value
Plasma DNA (for each increase of 1 GE/ml)	1.001	1.001 to 1.002	< 0.001	1.001	1.001 to 1.002	< 0.001
						
Plasma DNA for each increase of 500 GE/ml)	1.756	1.314 to 2.347	< 0.001	1.359	1.125 to 2.350	< 0.01
						
6-h lactate (for each increase of 1 mmol/l)	1.348	0.912 to 1.631	NS	1.115	0.765 to 1.530	NS
						
Age (for each increase of one year)	1.031	0.899 to 1.041	NS	1.376	0.865 to 1.240	NS
						
Admission glucose (for each increase of 10 mg/dl)	1.019	0.833 to 1.282	NS	1.012	0.780 too 1.320	NS

**Figure 1:**
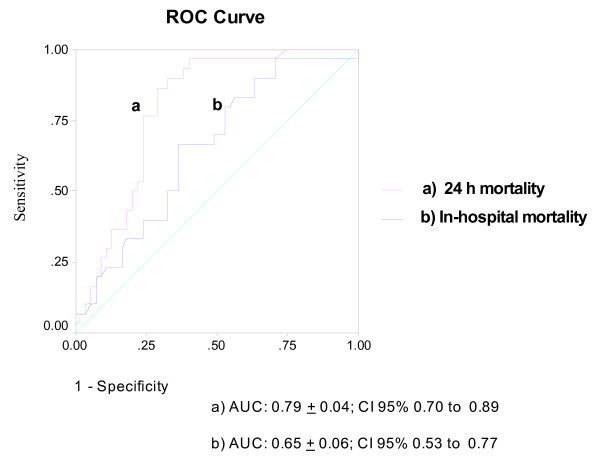
**Receiver operating characteristics curve for plasma DNA concentrations and 24-hour and in-hospital mortality**. The best cut-off value of plasma DNA for 24-hour mortality was 4,340 GE/ml (sensitivity 76%, specificity 83%), and for in-hospital mortality was 3,485 GE/ml (sensitivity 63%, specificity 69%).

**Figure 2:**
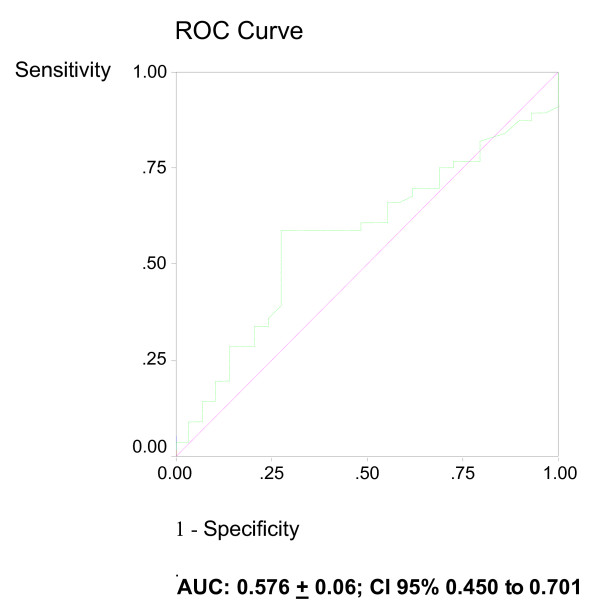
**Receiver operating characteristics curve for six-hour serum lactate concentrations and 24-hour and in-hospital mortality**. The best cutoff value of six-hour lactate in predicting 24-hour mortality was 7.1 mmol/l, with a sensitivity of 64%, specificity of 61%.

ROC curves were calculated for the use of plasma DNA as a predictor of 24-hour and in-hospital mortality and for lactate clearance to predict 24-hour mortality. The area under the ROC curves for plasma DNA to predict 24-hour mortality and in-hospital mortality were 0.796 (95% CI 0.701 to 0.890) and 0.652 (95% CI 0.533 to 0.770) (Figure 1). The area under the ROC curve for six-hour lactate concentration to predict 24-hour mortality was 0.576 (95% CI, 0.450 to 0.701) (Figure 2). The best cut-off value of plasma DNA at admission for 24-hour mortality was 4,340 GE/ml with a sensitivity of 76%, specificity of 83%, positive likelihood ratio of 2.41 (95% CI, 2.04 to 3.26) and correct classification rate of 73%. Regarding the secondary endpoint of in-hospital mortality, the best cut-off value of plasma DNA was 3,485 GE/ml with a sensitivity of 63%, specificity of 69%, positive likelihood ratio of 1.75 (95% CI, 1.44 to 2.35) and correct classification rate of 62%. The best cutoff value of six-hour lactate in predicting 24-hour mortality was 7.1 mmol/l, with a sensitivity of 64%, specificity of 61%, positive likelihood ratio of 1.32 (95% CI, 1.10 to 1.84) and correct classification rate of 57%.

## Discussion

A predictive test that would be applicable to comatose patients in the emergency department early after CPR is needed to help optimize the resuscitative efforts. This is the first prospective clinical study to evaluate the prognostic value of plasma DNA concentration on arrival at the emergency room in patients with out-of-hospital cardiac arrests. Our study shows that high plasma DNA concentration is associated with both 24-hour and in-hospital mortality. A multiple logistic regression analysis showed that raised plasma DNA level was a strong independent predictor of 24-hour mortality and was also independently associated with overall hospital mortality.

The post-resuscitation period after cardiac arrest has been compared to a sepsis-like syndrome, with components of circulatory, cardiogenic, and distributive shock [15]. It has been shown that plasma DNA is a useful independent predictor of mortality and sepsis in intensive care patients [16,17]. A prognostic value has also been found in emergency department patients with sepsis [18]. Cell-free plasma DNA measured on admission to the intensive care unit was found to be a predictor of outcome in severe sepsis and septic shock patients included in the Finnsepsis Study Group [19]. As current evidence suggests that the pathophysiology of post-cardiac arrest shock is very similar to that of patients with septic shock, we hypothesized that DNA concentrations at hospital admission might also predict mortality in patients in the immediate post-arrest period. The mechanisms underlying this period probably involve a whole-body ischemia and reperfusion instability that triggers the inflammatory cascade activation similar to that seen in severe sepsis. Plasma DNA is likely to be released from damaged and inflamed tissues, and in this context it might act as a marker of early outcome of patients with hypoxic-ischemic encephalopathy after cardiac arrest. We have demonstrated a role for plasma DNA as an early predictor of mortality in patients after cardiac arrest. Thus, the ability for rapid risk stratification of survival may allow clinicians to make more rational therapeutic decisions.

Moderate increases in plasma in plasma DNA may be associated with the chronic inflammatory response to atherosclerotic process which often occurs in elderly patients [28]. In our study there was no difference with respect to cardiovascular risk factors or chronic comorbidities except for diabetes within survivors and non-survivors patients and when entered into the logistic regression model for hospital mortality the adjusted odds ratio was not significant. Therefore it is likely that differences in plasma DNA levels in our study reflect the acute event of cardiac arrest rather than chronic illness.

Tissue hypo-perfusion during the early phase of post-cardiac arrest induces an increase in serum lactate because of anaerobic glycolisis. We have found that cell-free plasma DNA concentration at inclusion correlated significantly with initial lactate concentrations and maximum lactate concentrations within a 24-hour period, which may reflect the effect of tissue hypoxia on apoptotic or necrotic cell death. Effective lactate clearance which likely reflects improved tissue perfusion is associated with decreased mortality in severe sepsis and other critical-care patient populations [29,30]. Two studies have reported that post-cardiac arrest patients with more effective lactate clearance had improved survival [7,8]. Similarly, the current study revealed that lactate clearance at six hours was significantly higher in survivors compared to non-survivors at 24 hours, but we did not find this variable to be an independent predictor for early or late mortality when entered into the multivariable analysis. Further studies are required to establish the independent predictive value of effective lactate clearance after cardiac arrest.

An increase in plasma DNA concentration in critically ill patients may be also due to a decrease in clearance efficiency. The clearance mechanism of DNA from the circulation is poorly understood [31]. In mice, nucleotides are mainly cleared by liver [32]. Approximately 0.5 to 2% of circulating plasma DNA crosses the kidney barrier and is excreted into urine [33]. We found that serum urea or creatinine were not independently associated with plasma DNA concentrations, which is consistent with data from experimental studies. However, further investigations are required to understand the dynamics of plasma DNA removal in patients with impaired renal and hepatic function.

The current study has several methodological limitations. First, it is a single centre study for CPR after out-of-hospital cardiac arrest. Second, the majority of patients had unfavorable peri-arrest variables such as long downtime intervals and pulseless electric activity as presenting arrest rhythms. Third, some potential confounders like patient management at the emergency department and intensive care unit are difficult to control. In addition, some pre-analytical factors could have an impact on the results. To avoid contamination of the cell-free circulating plasma DNA measurements by residual white blood cells or platelets we used high-speed centrifugation at 16,000 g after storage, which almost completely eliminates cellular contamination in these assays. Opposing these limitations, the strengths of this study lie in the prospective design which includes a clearly defined patient sample, and the complete recording of premorbid, peri-arrest and immediate post-arrest variables. In addition, we measured lactate clearance as a marker of severity against which plasma DNA may be compared.

## Conclusions

In conclusion, our study results indicate that plasma DNA measurement on arrival at the emergency room may help physicians to estimate outcome in patients with cardiac arrest outside the hospital. In fact, plasma DNA concentration was a strong independent predictor for 24-hour mortality and was also independently associated with hospital mortality. A large prospective multicenter study is warranted to confirm the role of plasma DNA in outcome prediction after cardiac arrest and to validate the optimal plasma DNA cutoff levels regarding early and late mortality.

## Key messages

• The median plasma DNA concentration on arrival at the emergency department was two-fold higher in non-survivors at 24 hours compared to those in survivors following cardiac arrest.

• Plasma DNA concentration was a strong independent predictor for 24-hour mortality and was also independently associated with hospital mortality.

• Plasma DNA measurement on arrival at the emergency room may help physicians to estimate outcome in patients with cardiac arrest outside the hospital.

## Abbreviations

ADL: activities of daily life; CPR: cardiopulmonary resuscitation; COPD/emphysema: chronic obstructive pulmonary disease/emphysema; ED: emergency department; ER: emergency room; DNAR order: do not attempt resuscitation order; GCS: Glasgow Coma Scale; ICU: intensive care unit; PCR: Polymerase chain reaction; PEA: pulseless electrical activity; PVT: pulseless ventricular tachycardia; VF: ventricular fibrillation.

## Competing interests

The authors declare that they have no competing interests.

## Authors' contributions

FA, MM, EC, AQ and VL contributed to acquisition of the data. FA, RC, ELC and CM participated in the study design, coordination and statistical analysis. RC and CM performed the molecular analysis; FA, ELC and CM drafted the manuscript. All authors read and approved the final manuscript.
